# Infections with *Candidatus* Neoehrlichia mikurensis and Cytokine Responses in 2 Persons Bitten by Ticks, Sweden 

**DOI:** 10.3201/eid2108.150060

**Published:** 2015-08

**Authors:** Anna Grankvist, Lisa Labbé Sandelin, Jennie Andersson, Linda Fryland, Peter Wilhelmsson, Per-Eric Lindgren, Pia Forsberg, Christine Wennerås

**Affiliations:** University of Gothenburg, Göteborg, Sweden (A. Grankvist, J. Andersson, C. Wennerås);; Sahlgrenska University Hospital, Göteborg (A. Grankvist, J. Andersson, C. Wennerås);; Kalmar County Hospital, Kalmar, Sweden (L.L. Sandelin); Uppsala University, Uppsala, Sweden (L.L. Sandelin);; Linköping University, Linköping, Sweden (L. Fryland, P. Wilhelmsson, P.-E. Lindgren, P. Forsberg);; County Hospital Ryhov, Jönköping, Sweden (P.-E. Lindgren)

**Keywords:** Anaplasmataceae, Anaplasma phagocytophilum, Borrelia burgdorferi sensu lato, Candidatus Neoehrlichia mikurensis, bacteria, ticks, cytokine responses, immunocompetence, erythema, tick-borne infections, vector-borne infections, Sweden

## Abstract

The prevalence of *Candidatus* Neoehrlichia mikurensis infection was determined in 102 persons bitten by ticks in Sweden. Two infected women had erythematous rashes; 1 was co-infected with a *Borrelia* sp., and the other showed seroconversion for *Anaplasma phagocytophilum*. Both patients had increased levels of *Neoehrlichia* DNA and serum cytokines for several months.

*Candidatus* Neoehrlichia mikurensis is a tick-borne pathogen found in Europe and Asia (*1*). It causes an infectious disease in immunocompromised persons that is characterized by fever and thromboembolic events (*2*). In contrast, *Candidatus* N. mikurensis infection in immunocompetent hosts has been linked to asymptomatic infection (*3*), systemic inflammation with various symptoms (*4**,**5*), and possibly lethal infection (*6*). Knowledge regarding the capacity of *Candidatus* N. mikurensis to cause disease in immunocompetent persons is still limited. The purpose of this study was to investigate the prevalence, rate of co-infections, clinical picture, and cytokine response to *Candidatus* N. mikurensis infection in immunocompetent patients participating in the Tick-Borne Diseases Study (Technical Appendix).

## The Study

The study was approved by the Ethics Committees of Linköping University (M132-06), and Åland Health Care (2008-05-23). DNA was robot-extracted (MagNA Pure Compact Extraction Robot; Roche, Basel, Switzerland) from 400 µL of EDTA-plasma (Nucleic Acid Isolation Kit I; Roche) and analyzed by using a real-time PCR specific for a 169-bp segment of the *groEL* gene of *Candidatus* N. mikurensis. Amplifications were performed in a 20-µL reaction mixture containing 1× FastStart Taqman Probe Master (Roche), 1 µmol/L of each primer (5′-CGG AAA TAA CAA AAG ATG GA-3′; 5′- ACC TCC TCG ATT ACT TTA G-3′), 100 nmol/L of probe (5′-6FAM-TTG GTG ATG GAA CTA CA-MGB-3′), and 4 µL of DNA template. Real-time PCR was performed by using Rotorgene 6000 (QIAGEN, Hilden, Germany). Reaction conditions were 95°C for 10 min, followed by 45 cycles at 95°C for 15 s, and a final cycle at 54°C for 1 min. A synthetic plasmid containing the 169-bp sequence cloned into a pUC57 vector (Genscript, Piscataway, NJ, USA) was used to estimate bacterial gene copy numbers. Positive samples were verified by using a pan-bacterial PCR specific for the 16S rRNA gene (Technical Appendix). All PCR products were sequenced after electrophoresis on 2% agarose gels and analyzed by using an ABI PRISM 3130 Genetic Analyzer (Life Technologies Europe BV, Bleiswijk, the Netherlands). Obtained DNA sequences were edited and further analyzed by using the GenBank BLAST program (http://blast.ncbi.nlm.nih.gov/Blast.cgi) and Ripseq mixed software (Isentio, Palo Alto, CA, USA).

Patient serum samples were analyzed for antibodies against *Borrelia burgdorferi* sensu lato by using the RecomBead *Borrelia* IgM and IgG Kit (Mikrogen Diagnostik, Neuried, Germany). Samples were analyzed for IgG against *Anaplasma phagocytophilum* by using the *A. phagocytophilum* IFA IgG Kit (Focus Diagnostics, Cypress, CA, USA) and for 20 cytokines by using the Bio-Plex 200 System (Bio-Rad, Hercules, CA, USA).

A total of 102/3,248 study participants sought medical care during the 3-month study period and were further investigated. Their median age was 63 years (range 28–79 years) and 73 (72%) were women. All but 3 participants were immunocompetent (2 had cancer; 1 of them used methotrexate). *Candidatus* N. mikurensis DNA was detected in 2 (2.0%) of 102 patients, which is consistent with prevalences of 1.1% in China (*5*) and 1.6% in Poland (*3*).

Patient 1 was a healthy 68-year-old woman who lived on the island of Tjurkö, southeast of Sweden. She sought medical care on day 77 of the study because of a rash on her right breast. She reported being bitten by a tick in the same location 2 months earlier. The patient was given a diagnosis of erythema migrans, received phenoxymethylpenicillin (1 g, 3×/d for 10 days), and the rash disappeared.

Patient 2 was a 57-year-old woman who lived in Kalmar, Sweden. She had a history of allergy and was regularly taking aspirin. She had received treatment for Lyme borreliosis 8 years earlier. On day 65 of the study, she sought medical care because of a rash on her left breast. She reported being bitten by a tick in the same location 1.5 months earlier. The patient was also given a diagnosis of erythema migrans and received phenoxymethylpenicillin (1 g, 3×/d for 10 days).

Patient 1 had IgM against *Borrelia* outer surface protein C and pre-existing *Borrelia*-specific IgG titers that increased during the study (Table 1). Patient 2 was seronegative for *Borrelia* antigens throughout the study (Table 2). The rash of patient 1 may have been caused by co-infection with a *Borrelia* spp. Although there was no evidence of a *Borrelia* infection in patient 2, only 50% of *Borrelia* culture-positive patients with erythema migrans show development of specific antibodies (*7*). Moreover, early treatment for erythema migrans might abrogate the IgG response (*8*), although not always (*9*). Nevertheless, 20% of patients with erythema migrans show negative results for *Borrelia* DNA in the skin, which indicates that these rashes might be caused by other infectious agents (*10*). Our study indicates that an erythematous rash in persons bitten by ticks might not be caused by *Borrelia* spp. and might require treatment with doxycycline instead of penicillin.

**Table 1:** Evolution of *Candidatus* Neoehrlichia mikurensis gene copy numbers and antibody levels to *Borrelia burgdorferi* sensu lato complex and *Anaplasma phagocytophilum* for patient 1, Sweden

Characteristic	Days after inclusion in study		
	0	77	169
Clinical manifestations	None	Rash on right breast	None
*Candidatus* N. mikurensis DNA in plasma, gene copies/mL	0	2,200	2,000
Serum *B. burgdorferi* sensu lato IgM (points)*	Negative (4)	Positive (12)	Positive (8)
*B. burgdorferi* sensu lato antigens			
Positive reactivity	None	OspC, p100	OspC
Borderline reactivity	None	None	None
Serum *B. burgdorferi* sensu lato IgG (points)†	Positive (13)	Positive (16)	Positive (16)
*B. burgdorferi* sensu lato antigens			
Positive reactivity	P100, VlsE, p58	P100, VlsE, p58, OspC	P100, VlsE, p58, OspC
Borderline reactivity	OspC	None	None
Serum *A. phagocytophilum* IgG (1:64)‡	++	++	++
Serum *A. phagocytophilum* IgG (1:256)§	+	+	+

*Reactivity to either outer surface protein C (OspC) alone or to 2 antigens was required for a positive IgM response. †Reactivity to >2 antigens was required for a positive IgG response. Full reactivity to an antigen is indicated by 4 points. ‡Every serum sample was tested at a dilution of 1/64. ++, strongly positive; +, positive. §Samples that showed a positive (+) result were further tested at dilutions of 1:128 and 1:256.

**Table 2:** Evolution of *Candidatus* Neoehrlichia mikurensis gene copy numbers and antibody levels to *Borrelia burgdorferi* sensu lato complex and *Anaplasma phagocytophilum* for patient 2, Sweden

Characteristic	Days after inclusion in study		
	0	65	98
Clinical manifestations	None	Rash on left breast	None
*Candidatus* N. mikurensis DNA in plasma, gene copies/mL	0	260	1300
Serum *B. burgdorferi* sensu lato IgM (points)*	Negative (0)	Negative (2)	Negative (1)
*B. burgdorferi* sensu lato antigens			
Positive reactivity	None	None	None
Borderline reactivity	None	p39	p39
Serum *B. burgdorferi* sensu lato IgG (points)†	Negative (0)	Negative (4)	Negative (4)
*B. burgdorferi* sensu lato antigens			
Positive reactivity	None	VlsE	VlsE
Borderline reactivity	None	None	None
Serum *A. phagocytophilum* IgG (1:64)‡	±	+	++

*Reactivity to either outer surface protein C alone or to 2 antigens was required for a positive IgM response. †Reactivity to >2 antigens was required for a positive IgG response. ‡Every serum sample was tested at a dilution of 1/64. ±, weakly positive; +, positive; ++, strongly positive.

Patient 1 had pre-existing IgG against *A. phagocytophilum* that remained unchanged (Table 1). Patient 2 had borderline levels of IgG against *A. phagocytophilum* on day 0, which increased successively on days 65 and 98 (Table 2). This seroconversion may have resulted from cross-reactivity with *Candidatus* N. mikurensis, which was previously reported for an immunocompetent patient from Switzerland (*4*). Relatively high rates of seropositivity to *A. phagocytophilum* in Sweden (*11**,**12*) might be caused by cross-reactive antibodies because *Candidatus* N. mikurensis is common in ticks in Sweden, in contrast to *A. phagocytophilum* (*13*).

**Figure 1:**
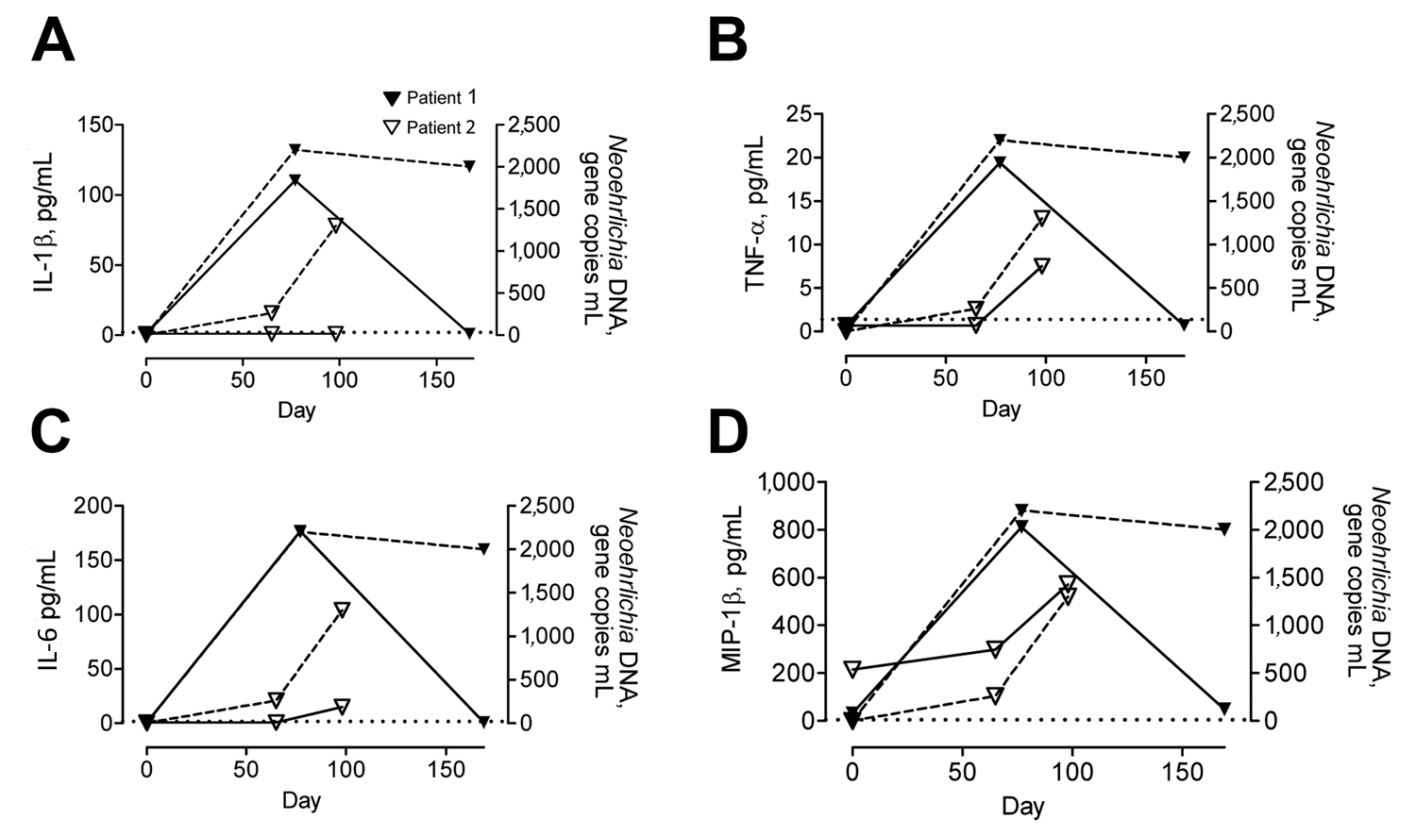
Proinflammatory cytokines in 2 patients infected with *Candidatus* Neoehrlichia mikurensis, Sweden. Concentrations of cytokines A) interleukin-1β (IL-1β), B) tumor necrosis factor-α (TNF-α), C) interleukin-6 (IL-6), and D) macrophage inflammatory protein-1β (MIP-1β) were measured in serum of patient 1 on days 0, 77, and 169 and in serum of patient 2 on days 0, 65, and 98. A rash developed in patient 1 on day 77 and in patient 2 on day 65. Dashed lines indicate levels of *Neoehrlichia* DNA in plasma for both patients. Dotted lines indicate detection limit for each cytokine.

**Figure 2:**
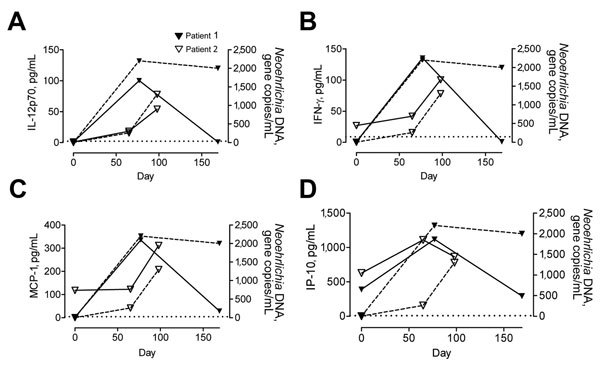
Th1 cytokines in 2 patients infected with *Candidatus* Neoehrlichia mikurensis, Sweden. Concentrations of cytokines A) interleukin-12p70 (IL-12p70), B) interferon-γ (IFN-γ), C) monocyte chemoattractant protein-1 (MCP-1) (C), and D) IFN-γ−induced protein 10 (IP-10) were measured in serum of patient 1 on days 0, 77, and 169 and in serum of patient 2 on days 0, 65, and 98. Dashed lines indicate levels of *Neoehrlichia* DNA in plasma for both patients. Dotted lines indicate detection limit for each cytokine.

Both patients showed increased serum levels of cytokines, which appeared to mirror the numbers of *Candidatus* N. mikurensis gene copies (Figures 1, 2; Technical Appendix Figure). Cytokine levels for patient 1 were maximum on day 77 and returned to reference levels on day 167. All cytokines, except for interferon-γ−induced protein 10, reached maximum levels on day 98 for patient 2. The cytokines were selected because systemic inflammation (Figure 1) with neutrophilia (Technical Appendix) is typical of neoehrlichiosis in immunocompromised patients (*2*). In addition, a Th1-like immune response (Figure 2) is presumably required to eliminate an intracellular pathogen, such as *Candidatus* N. mikurensis. However, the cytokine response of patient 1 may in part have been caused by *Borrelia* spp. (*14*).

## Conclusions

*Candidatus* N. mikurensis DNA was detected in the blood of both patients for >1 and 3 months, respectively. Similarly, a healthy person in Poland showed a positive result for *Candidatus* N. mikurensis twice in a 4-month period (*3*). This finding suggests that *Candidatus* N. mikurensis infections persist for a long time or that frequent reinfections occur. Prolonged carriage seems more probable in view of the common occurrence of neoehrlichiosis during winter among immunocompromised patients (*2*); immunosuppressive therapy might reactivate such infections. An analogous finding was reported in a dog, which was believed to have been a chronic carrier of *Candidatus* N. mikurensis; infection became symptomatic when immune defenses were compromised by surgery (*15*).

In conclusion, an erythematous rash in a person bitten by a tick can be caused by *Candidatus* N. mikurensis, rather than by *Borrelia* spp. Moreover, immunocompetent persons may be infected by *Candidatus* N. mikurensis for unexpectedly long periods, even after symptoms have disappeared. Patients scheduled to receive immunosuppressive treatment, and who live in *Candidatus* N. mikurensis–endemic areas should be screened for this pathogen before beginning therapy.

**Technical Appendix.** Tick-borne disease study design and pan-bacterial PCR.
