# Supplementary material for: Infections with Candidatus Neoehrlichia mikurensis and Cytokine Responses in 2 Persons Bitten by Ticks, Sweden
Source: Emerg Infect Dis. 2015 Aug;21(8):1462–5. doi: 10.3201/eid2108.150060 (PMC4517700; doi:10.3201/eid2108.150060)
Supplement: Supplementary file 1 — Technical Appendix. Tick-borne disease study design and pan-bacterial PCR. [file 15-0060-Techapp-s1.pdf]

Article DOI: http://dx.doi.org/10.3201/eid2108.150060

Infections with *Candidatus* Neoehrlichia mikurensis and Cytokine Responses in 2 Persons Bitten by Ticks, Sweden

Technical Appendix

Tick-Borne Diseases Study Design

The ongoing Tick-Borne Diseases Study was initiated in 2007 to investigate the prevalence of pathogens in ticks that have fed on humans and the risk for developing tick-borne disease after a bite by an infected tick. Blood samples from persons residing in Sweden or the Åland Islands, Finland, who were bitten by ticks; health questionnaires; and ticks that had bitten the persons were collected at the 34 primary health care centers that participated in the study (*1*–*3*). At the time of inclusion, the participant completed a questionnaire containing questions about past and current medical history and data about the current tick bite. Tick(s) that had bitten participants were provided, and blood samples (plasma and serum stored in EDTA) were obtained.

At a follow-up visit 3 months later, the participant completed a second questionnaire containing questions about new tick bites during the study period, general health conditions, any signs or symptoms possibly associated with tick-borne disease; donated any additional ticks that had bitten the participant since the first visit; and provided blood samples. Study participants were asked to obtain health care when needed during the 3-month study period. Additional blood samples were collected at these visits and treatment with antimicrobial drugs was initiated at the discretion of the physician if tick-borne disease was suspected. During 2008–2012, a total of 3,248 persons participated in the Tick-Borne Diseases Study. In the present study, 102 participants who sought medical care at their health care center during the study were investigated.

Pan-bacterial PCR

For amplification of a segment of the bacterial 16S rRNA gene, the forward primer SSU1 5′-CGG CAG GCC TAA CAC ATG CAA GTC G-3′ and the reverse primer 806R, 5′-GGA CTA CCA GGG TAT CTA AT-3′ were used, which are complementary to conserved regions in the 5′ half of the 16S rRNA gene and yield a fragment of 766 bp between

nucleotide positions 41 and 806 for *Escherichia coli*. The amplification procedure was performed as described (*4*). For sequencing, PCR products of the expected size (766 bp) were cut out of the gel, purified by using the Qiaquick Gel Extraction Kit (QIAGEN, Göteborg, Sweden), and cycle-sequenced by using the ABI PRISM Big Dye Terminator Cycle Sequencing Ready Reaction Kit, Version 1.1 (Life Technologies Europe BV, Bleiswijk, the Netherlands) in 1 direction way with the reverse primer (806R).

References

1. Wilhelmsson P, Fryland L, Börjesson S, Nordgren J, Bergström S, Ernerudh J, et al. Prevalence and diversity of *Borrelia* species in ticks that have bitten humans in Sweden. J Clin Microbiol. 2010;48:4169–76. PubMed http://dx.doi.org/10.1128/JCM.01061-10

2. Fryland L, Wilhelmsson P, Lindgren PE, Nyman D, Ekerfelt C, Forsberg P. Low risk of developing *Borrelia burgdorferi* infection in the south-east of Sweden after being bitten by a *Borrelia burgdorferi*-infected tick. Int J Infect Dis. 2011;15:e174–81. PubMed http://dx.doi.org/10.1016/j.ijid.2010.10.006

3. Wilhelmsson P, Lindblom P, Fryland L, Nyman D, Jaenson TG, Forsberg P, et al. *Ixodes ricinus* ticks removed from humans in northern Europe: seasonal pattern of infestation, attachment sites and duration of feeding. Parasit Vectors. 2013;6:362. PubMed http://dx.doi.org/10.1186/1756-3305-6-362

4. Welinder-Olsson C, Dotevall L, Hogevik H, Jungnelius R, Trollfors B, Wahl M, et al. Comparison of broad-range bacterial PCR and culture of cerebrospinal fluid for diagnosis of community-acquired bacterial meningitis. Clin Microbiol Infect. 2007;13:879–86. PubMed http://dx.doi.org/10.1111/j.1469-0691.2007.01756.x

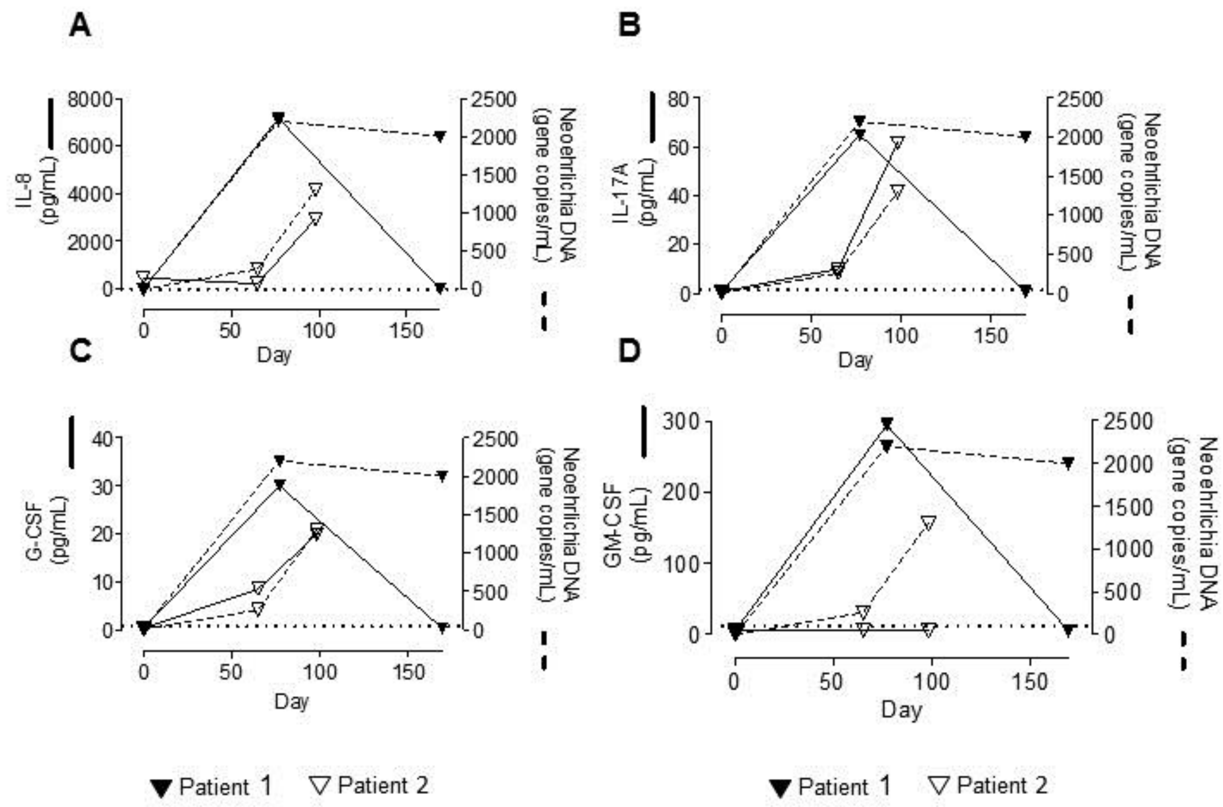

Technical Appendix Figure. Cytokines essential for recruitment or maturation of neutrophils in 2 patients infected with *Candidatus* Neoehrlichia mikurensis, Sweden. Concentrations of cytokines A) interleukin-8 (IL-8), B) interleukin-17A (IL-17A), C) granulocyte colony-stimulating factor (G-CSF), and D) granulocyte macrophage–colony-stimulating factor (GM-CSF) were measured in serum of patient 1 on days 0, 77, and 169 and in serum of patient 2 on days 0, 65, and 98. Levels of *Neoehrlichia* DNA in plasma are shown for both patients. Dotted lines indicate detection limit for each cytokine.
